# Superior ophthalmic vein cutdown and craniotomy for sylvian vein access in the treatment of a bilateral spontaneous indirect carotid-cavernous fistula

**DOI:** 10.3171/2025.7.FOCVID25103

**Published:** 2025-10-01

**Authors:** Ross Greenberg, James Feghali, Risheng Xu, Christopher M. Jackson, Justin M. Caplan, Nicholas R. Mahoney, Judy Huang, Rafael J. Tamargo, L. Fernando Gonzalez

**Affiliations:** Departments of 1 Neurosurgery and; 2 Ophthalmology, Johns Hopkins University School of Medicine, Baltimore, Maryland

**Keywords:** carotid-cavernous sinus fistula, embolization, cerebral arteries

## Abstract

The authors discuss the first reported case of bilateral spontaneous indirect carotid-cavernous fistula (CCF) treated by a hybrid open-endovascular approach bilaterally. A 74-year-old man presented to the emergency department following 1 month of progressive right-eye proptosis with increased intraocular pressure. Cerebral angiography confirmed bilateral indirect type D CCF. Attempts to access the cavernous sinus through the petrosal sinuses were unsuccessful. The right-sided CCF was successfully treated via surgical exposure of the superior ophthalmic vein for endovascular deployment of coils and liquid embolic agent. The left-sided CCF was similarly treated after gaining cavernous sinus access via craniotomy to catheterize the left sylvian vein.

The video can be found here: https://stream.cadmore.media/r10.3171/2025.7.FOCVID25103

## Transcript

### 0:20 Introduction and Patient Presentation.

We present the case of a bilateral spontaneous indirect carotid-cavernous fistula where both superior ophthalmic vein cutdown and craniotomy for sylvian access were utilized for transvenous access and endovascular treatment

A 74-year-old man presented to the emergency department with 1 month of progressive and painless right eye swelling and redness that acutely worsened over 2 weeks. Extraocular movements were globally restricted on the right with ptosis. Intraocular pressure was 35 mm Hg on the right and 10 mm Hg on the left. Visual acuity was 20/80 on the right and 20/40 on the left with full visual fields to confrontation. CT angiogram showed early enhancement of bilateral cavernous sinuses and a dilated right superior ophthalmic vein consistent with a carotid-cavernous fistula. The patient was started on pressure-lowering eyedrops and admitted to the neurocritical care unit in preparation for digital subtraction angiography and treatment.

### 1:17 Diagnostic Cerebral Angiography.

Left femoral artery along with right femoral vein access were obtained in anticipation of transvenous embolization. Snapshots from the angiogram are shown with AP (anteroposterior) projections in the left panels and lateral projections in the right panels. Injections in the right ICA (internal carotid artery), shown in panel A, and right ECA (external carotid artery), shown in panel C, demonstrate early filling of the right cavernous sinus, with a dilated right superior ophthalmic vein (which is denoted by the wide blue arrow); this is consistent with the patient’s right eye signs and symptoms. The right ECA injection also demonstrated early venous drainage into the left cavernous sinus. The left ICA and ECA injections shown in panels B and D, respectively, demonstrate early filling of the left cavernous sinus but with retrograde sphenoparietal sinus drainage (denoted by the narrow blue arrow) and sylvian vein drainage. Both fistulas represent a Barrow classification type D CCF (carotid-cavernous fistula)^1,2^ given contributions from dural branches of both the internal and external carotid arteries. Transvenous embolization of the right fistula was attempted, but access to the cavernous sinus was unsuccessful. No clearly patent inferior petrosal sinus could be identified, and attempts to navigate through the presumed location of the inferior petrosal sinus with a microcatheter were unsuccessful. The procedure was uncomplicated with a stable postoperative exam.

### 2:37 Stage 1: Direct Right SOV Cutdown for Transvenous CCF Embolization.

Given failure of a traditional transfemoral approach and the noted venous drainage pattern, stage 1 embolization of the right fistula utilizing direct right superior ophthalmic vein cutdown with the aid of oculoplastic surgery was planned.^3,4^

The endovascular neurosurgery team obtained right femoral arterial access, and diagnostic cerebral angiography showed no major changes in the configuration of the bilateral fistula from angiography the day prior. The oculoplastic surgery team then exposed the right superior ophthalmic vein in the endovascular suite. Cutdown consisted of an incision in the medial upper eyelid crease followed by careful opening of the orbicularis oculi and blunt dissection of the orbital septum and medial fat pad to identify the vein. The superior ophthalmic vein was isolated with a rubber vessel loop, shown in the left panel, and subsequently accessed with an 18-gauge angiocath, as shown in the right panel.

A venogram, shown in panel A, confirmed that the catheter is indeed in the superior ophthalmic vein, and this demonstrated opacification of the right cavernous sinus as well as a single very small intercavernous connection to the left cavernous sinus with faint opacification of the sphenoparietal sinus and sylvian vein on the left. Attempts to catheterize the left cavernous sinus through the intercavernous connection were unsuccessful despite multiple attempts. The right fistula was embolized by deploying coils and liquid embolic into the right cavernous sinus. Embolic did not travel through the intercavernous connection to the left side, likely since this connection was a high-resistance channel, with other lower-resistance paths such as the superior ophthalmic vein still patent. Shown in panel B, a postembolization right common carotid artery injection confirmed complete obliteration of the right fistula. Left-sided internal carotid and external carotid artery injections additionally confirmed no new arterial feeders to the right fistula. The patient was transferred back to the neurocritical care unit with a stable exam.

### 4:31 Stage 2: Left Pterional Craniotomy for Direct Sylvian Vein Access and Transvenous CCF Embolization.

Treatment of the left fistula in stage 2 was undertaken 4 days later. Due to inaccessibility of the left cavernous sinus from the petrosal sinuses or from the right cavernous sinus, in addition to the lack of a dilated left superior ophthalmic vein and presence of multiple arterial feeders, a left pterional craniotomy for direct sylvian vein access^5^ and transvenous embolization were planned.

Here we have the preoperative CT angiogram. Scrolling through, a prominent left sylvian vein is evident. This vessel was the target vessel to be accessed with the aid of frameless stereotactic navigation.

The patient was positioned supine with the head turned to the right. The skull was held in place with a Mayfield fixation device. The scalp was marked for a standard incision used in a pterional craniotomy. Fiducials for navigation were present on various landmarks on the face and scalp.

At this point, the left-sided pterional craniotomy had been completed. The dura had been retracted anteriorly with 4-0 Nurolon sutures, and the distal aspect of the sylvian fissure had been opened. This image reveals the arterialized left sylvian vein marked with the yellow star. There is also obvious arterialization of the frontal veins marked by the red star. Note the veins’ red and engorged appearance.

The left sylvian vein is now being entered with a 20-gauge needle followed by the introduction of an angiocath. A microwire is being introduced in order to advance the angiocath further into the vessel. The wire is being removed to facilitate closure and anchoring of the angiocath.

The venous catheter is now being secured in place with a 5-mm temporary aneurysm clip. A nylon suture is being used to tie a relatively loose knot in the scalp under the catheter hub. A subsequent knot is being tied over the hub to secure it to the scalp while the assistant holds the catheter in place with a smooth pickup. Patency and access was confirmed by gentle suction on the catheter hub throughout the procedure. A check-flow valve is being placed onto the angiocath hub. After this, the scalp was temporarily reapproximated with skin staples, and the patient was transported safely to the angiography suite.

In panel A, a venogram from a left sylvian vein injection demonstrates left cavernous sinus opacification. The fistula was obliterated by deposition of coils and liquid embolic in the cavernous sinus. In panel B, postembolization internal carotid and external carotid artery injections demonstrate complete obliteration with a snapshot of the final cast. The patient was then transported back to the OR for standard closure after removal of the angiocath. Postoperative exam was stable, and the patient was discharged home on postoperative day 4.

### 7:13 Postoperative Follow-Up.

The patient was seen in clinic 2 weeks postoperatively with normalized intraocular pressures and improved extraocular eye movements. Four months later, he remains asymptomatic with a normal ophthalmological exam.

### 7:24 Conclusion.

In conclusion, this constitutes a uniquely reported case of a bilateral spontaneous indirect carotid-cavernous fistula treated by a hybrid approach on both sides by accessing the right superior ophthalmic vein deep to the eyelid and the left sylvian vein through a craniotomy. In instances where traditional endovascular routes are not feasible, alternative vascular access via open techniques based upon review of venous drainage patterns can be safe and effective for treating carotid-cavernous fistulas.

## Disclosures

Dr. Huang reported stock ownership in Longeviti during the conduct of the study. Dr. Gonzalez reported being a consultant for Medtronic outside the submitted work.

## Author Contributions

Primary surgeon: Gonzalez. Assistant surgeon: Feghali, Xu, Mahoney. Editing and drafting the video and abstract: Gonzalez, Greenberg, Feghali. Critically revising the work: Gonzalez, Greenberg, Feghali, Xu, Huang. Reviewed submitted version of the work: Gonzalez, Greenberg, Feghali, Xu, Jackson, Caplan, Huang, Tamargo. Approved the final version of the work on behalf of all authors: Gonzalez. Supervision: Gonzalez, Huang.

## Supplemental Information

### Patient Informed Consent

The necessary patient informed consent was obtained in this study.
